# Efficacy of curcumin gel as an adjunct to scaling and root planing on salivary procalcitonin level in the treatment of patients with chronic periodontitis: a randomized controlled clinical trial

**DOI:** 10.1186/s12903-023-03512-y

**Published:** 2023-11-19

**Authors:** Reham Abdel-Fatah, Bassant Mowafey, Azza Baiomy, Samah Elmeadawy

**Affiliations:** 1 Oral Medicine, Periodontology, Diagnosis, and Oral Radiology Department, Faculty of Dentistry, Mansoura University, Mansoura, 35516 Egypt; 2 Clinical Pathology Department, Faculty of Medicine, Mansoura University, Mansoura, Egypt

**Keywords:** Chronic periodontitis, Curcumin, Scaling, Root planing, Procalcitonin, Randomized controlled clinical trial

## Abstract

**The aim of the study:**

To evaluate the effect of curcumin gel combined with scaling and root planing (SRP) on salivary procalcitonin in periodontitis treatment.

**Materials and methods:**

seventy patients were selected from the Department of Oral Medicine and Periodontology, Faculty of Dentistry, Mansoura University, and sixteen patients were excluded. Patients in groups II and III included stage II grade A periodontitis. The participants were classified into three groups: group I as a negative control group (individuals with healthy gingiva), group II (SRP) were treated with SRP, and group III (curcumin gel) which was applied weekly for four weeks after SRP. Clinical indices (plaque index (PI), gingival index **(GI)**, clinical attachment level (CAL), and probing depth (PD)) and saliva samples for procalcitonin (PCT) assessment using an enzyme-linked immunosorbent assay (ELISA) test were collected and measured at both baselines and after six weeks.

**Results:**

This randomized controlled clinical trial **registered on ClinicalTrials.gov (NCT05667376) and first posted at 28/12/2022** included Fifty-four patients (20 male; 34 female). Regarding the age and sex distribution, there was no statistically significant difference between the three studied groups (p > 0.05). There was no significant statistical difference regarding PI, GI, PPD, and CAL between group II and group III at baseline p (> 0.05). However, there was a significant statistical difference regarding the clinical parameters at baseline of both group II and group III as compared to group I (p ≤ 0.05). At six weeks after treatment, group III showed greater improvement in the PI, PD, and CAL as opposed to group II (p ≤ 0.05). Regarding PCT values, at baseline, there wasn’t a statistically significant difference between group II and group III (p > 0.05). However, there was a significant statistical difference between group II, group III, and group I (p ≤ 0.05). At six weeks after treatment, there was a statistically significant decrease in PCT levels of both group II and III (p ≤ 0.05).

**Conclusion:**

The application of curcumin gel was found to have a significant effect on all clinical indices as opposed to SRP.

## Introduction

Periodontal disease is a disease of periodontium characterized by an inflammatory response in the tooth-supporting structures. This can destroy the periodontal ligament surrounding teeth and alveolar bone and will lead to tooth mobility and eventual tooth loss [1, 2].

The pathogenesis of periodontal disease has been described as a breakdown in the periodontium due to changes in the host’s inflammatory immune response. The bacterial by-products in biofilm result in inflammatory reactions and subsequent release of different cytokines and enzymes with the capacity to initiate tissue destruction and breakdown [3, 4].

The primary objective of periodontal treatment is to reduce and eliminate bacterial load and improve clinical manifestations using a combination of surgical and non-surgical treatment modalities. Despite being the most used non-surgical procedure, SRP fails to overcome the bacterial biofilm in deep pockets and areas that are challenging to access conventionally. This has led to the evolution of the concomitant use of antibacterial agents as a method of local drug delivery [5, 6].

These antimicrobial agents can compensate for technical limitations, prevent early bacterial recolonization, and provide an opportunity for clinical improvements. However, these agents have been associated with side effects and high costs. Therefore, to overcome these drawbacks, research has been conducted on the use of natural herbal products including herbal formulations such as turmeric and curcumin [7].

Used as a spice around the world and found in traditional eastern medicine, curcumin is one of the most extracted polyphenols from the rhizomes of the Curcuma longa plant. The U.S. Food and Drug Administration (FDA) considers curcumin “a safe compound” [8]. Its anti-carcinogenic, antioxidant, anti-inflammatory, antiviral, and anti-microbial properties have been demonstrated in various conditions and experiments [9], including the treatment of periodontitis [10–12]. In periodontitis, curcumin has exhibited anti-inflammatory and antimicrobial properties. The anti-inflammatory action of curcumin involves lowering inflammatory infiltrate [10, 12], pro-inflammatory cytokines [10], osteoclastogenesis, inflammatory bone resorption [10, 13], and nuclear factor-kappa B (NF-KB) activation [12].

In addition, the tested clinical trials demonstrate that local [14–16] and systemic application of curcumin can improve the results of SRP in the treatment of periodontitis. However, Local administration allows higher concentrations of the active ingredients in the diseased site and prevents possible side effects of systemic usage [17]. It has been established that the inflammatory biomarkers in different biologic fluids and their levels are beneficial indicators for the evaluation of inflammatory process activity. Therefore, the analysis of saliva and gingival crevicular fluid (GCF) is beneficial for the assessment of the existing periodontal conditions [18–21].Thus, the recent usage of biomarkers sensitive to bacterial infection to aid in clinical judgment is a promising approach for reducing or limiting antibiotic usage [22].

Periodontitis adversely influence numerous systemic diseases, and affect above 50% of the population all over the world such as preterm birth, cardiovascular disease (CVD), and metabolic syndrome. the periodontitis progression may be linked with the dysregulation of some pro-inflammatory mediators released in the bloodstream, such as metalloproteases, prostaglandins, interleukins, and high-sensitive c-reactive proteins, which could increase chronic risk of systemic inflammation, endothelial dysfunction, and CVD through a specific oxidative stress pathway. There is emergent concern in evaluating the influence of periodontal treatment on early biomarkers related to early CVD risk and assessing the influence of biomarker levels on the longstanding effectiveness of periodontal treatment. One of the biomarkers of early CVD risk, it has been established that the N-terminal pro-Btype natriuretic peptide (NT-proBNP), linked in the early diagnosis, risk stratification, and follow-up outcomes of patients with CVD and heart failure. NT-proBNP is a fragment secreted by cardiac ventricles myocytes in response to the heart volume expansion or pressure load [23–26].

Galectins also are a family of beta-galactoside-binding proteins released in fibroblast, epithelial cells, and during active stages of inflammation. Galectin-3 which is systemically released from fibroblast, and macrophage on active inflammation sites, has been proven to be involved during cell adhesion, inflammatory response, tissue fibrosis, and first immunity response. Galectin-3 is also associated with the early stages of certain systemic diseases such as endothelial dysfunction, coronary heart disease (CHD), and heart failure [27–29].

These inflammatory biomarkers include PCT which has been used on a large scale as a potential reliable diagnostic biomarker for several systemic infectious diseases such as septic shock, sepsis, and meningitis. Moreover, it has been found that routine analysis of PCT levels appears to be valuable in the early recognition and diagnosis of inflammatory systemic diseases [30].The pro-inflammatory cytokines released at the periodontally diseased tissues can stimulate and further activate the liver cells to release the acute phase proteins [31]. These acute-phase proteins include; C-reactive protein (CRP) and PCT which are significant biomarkers essential for the diagnosis of different forms of periodontitis [32, 33] as periodontitis has been associated with the increased serum levels of these proteins.

The PCT peptide is an established serum marker of inflammation with the bacterial LPS being a potent inducer of it in the systemic circulation. Additionally, PCT has been evaluated with several biomarkers of sepsis and it has been authorized to be more specific with higher prognostic values than CRP [32, 34] thus, the bacterial infection diagnosis via PCT use has become ever-increasing [34, 35]. PCT has been considered a potentially harmful inflammatory mediator because it has a vasodilatory response (like Calcitonin Gene-Related Peptide) and encourages releasing of inflammatory mediators such as interleukin (IL)-6, tumor necrosis factor (TNF), coagulation proteins, and acute phase proteins (e.g., CRP ). [34].

Furthermore, US-FDA acknowledged serum PCT to guide antibiotic therapy [36] as it is a beneficial biomarker in several forms of periodontitis [37, 38]. Moreover, the sensitivity of PCT in bacteremia induced by non-surgical periodontal therapy (NSPT) has needed to be assessed as local PCT production is stimulated by periodontal diseases while NSPT can reduce inflammatory changes.

Additionally, the serum PCT levels can aid in infectious disease diagnosis, prognosis, and also their conversion to systemic inflammation. Furthermore, PCT level can be evaluated to determine the focal infection elimination and the applied therapy success as serum concentration of PCT has been found to increase in several inflammatory conditions so it is a beneficial predictor in such cases as sepsis, bacterial meningitis, bacteremia, bacterial super-infection, and acute pancreatitis [39]. Therefore, this study was conducted to evaluate the efficacy of curcumin gel as a local drug delivery system adjunct to SRP in the treatment of periodontitis. Furthermore, we aimed to evaluate the curcumin efficacy on salivary PCT levels in periodontitis patients.

## Materials and methods

### Study population

The randomized controlled clinical trial **registered on ClinicalTrials.gov (Identifier: NCT05667376 ) and first posted at 28/12/2022** included fifty-four participants selected from the Department of Oral Medicine and Periodontology Outpatient Clinic, Faculty of Dentistry, Mansoura University from January 2021 to May 2021. Thirty-six patients with periodontitis (stage II grade A) and eighteen healthy volunteers (negative control group) participated in the study. The inclusion criteria were participants of both sexes, between the ages of thirty and fifty -five years old with periodontitis (stage II grade A). The exclusion criteria involved participants with known systemic diseases, pregnant and lactating patients, tobacco users (smoking /chewing), and history of antibiotic and periodontal therapy in the last three months, and participants who weren’t compliant with oral hygiene procedures.

### Pretreatment period

Participants were informed about the purpose of the study and screened for eligibility. They also were informed about the treatment received and the steps done, including non-surgical treatment and gel application with the associated risks, possible effects, and other treatment options available. The participants understood this explanation in broad terms according to the rules of the ethical committee of the Faculty of Dentistry, Mansoura University. Additionally, they acknowledged that they would be required to attend the periodic recall visits and that they are legally competent to give written informed consent before performing any required steps.

### Randomization and study groups

**Fig. 1:**
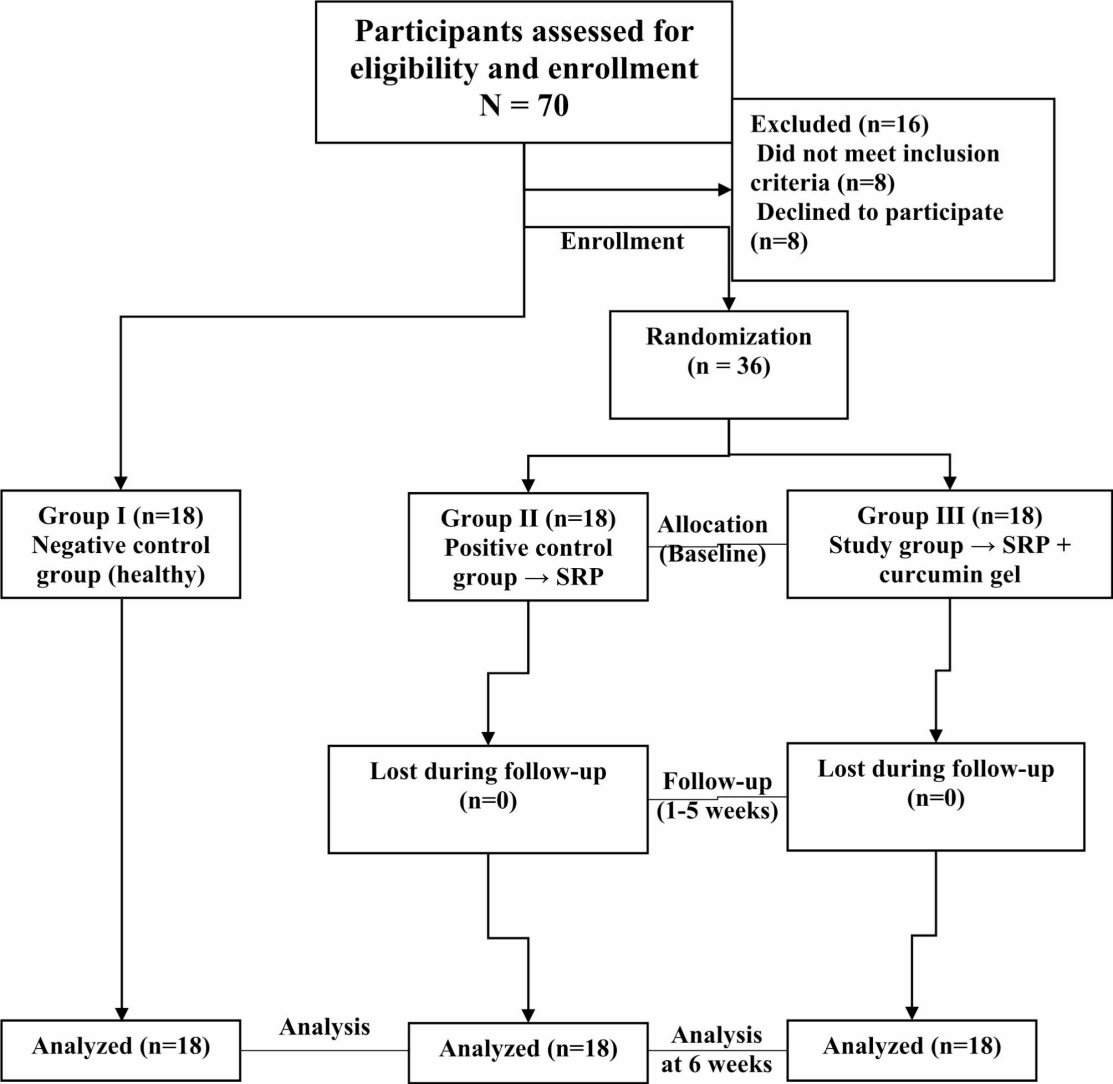
Study flowchart

The patients with periodontitis groups were randomly selected into either group II or III using the coin toss method. The participants with periodontitis were unaware of which group they were allocated to (study group or positive control group) as they were randomly assigned into either group II or group III using the coin toss method [40] by another masked examiner other than the operator. Hence, the study groups were classified into three groups as follows, a group I (negative control group): eighteen subjects with clinically healthy gingiva were selected to participate in the study as a reference group, those patients were given neither curcumin gel nor placebo gel, but they selected on the basis of the new classification system of periodontal and peri-implant diseases and conditions (patients with healthy gingiva on intact periodontium) [41]. Group II (positive control group): eighteen patients with periodontitis (stage II grade A) were selected and treated with non-surgical periodontal therapy (SRP) only, and group III (study group): eighteen patients with periodontitis (stage II grade A) were selected and treated with SRP followed by curcumin gel application (Fig. 1).

### Saliva sample collection

Saliva sample collection was accomplished using the swabbing method. It was performed by introducing a cotton pad into the mouth at the orifices of major salivary glands. The subjects were asked to chew the cotton pad to get soaked in the saliva. The saliva-soaked pad was removed and placed in a sterile test tube (sterile Eppendorf tube) and centrifuged at 3000 rounds per minute (RPM) for approximately 10 min and then kept frozen at -20°c till the time of analysis.

### Periodontal treatment

#### SRP procedures

At the beginning of treatment, the periodontitis patients were treated with SRP using both special tipped ultrasonic scalers and manual Gracey curettes until a smooth tooth/root surface was achieved. The SRP procedure was repeated once again (if necessary) and instructions were given to the patients to maintain good oral hygiene measures during the treatment period. Brushing and flossing techniques were taught to all participants using patient education videos and demonstrated on study models. No antibiotics or mouthwashes were prescribed during treatment. All patients were recalled weekly during the study period (which was carried out for six weeks) for reinforcement of oral hygiene measures and curcumin gel application inside the only selected periodontal pockets of group III as it was applied four times during the whole study period.

#### Curcumin gel preparation and application

The gel preparation was carried out in the Pharmacology Department, Faculty of Pharmacy, Mansoura University. The 2% curcumin gel was prepared by the method of simple dispersion (Table 1). Carbopol 940 was drenched in filtered water containing 0.2% w/v sodium benzoate overnight. Hydroxypropyl methylcellulose (HPMC) solution was mixed using a tissue homogenizer in propylene glycol.

**Table 1:** Formula to prepare 2%curcumin gel

Ingredients	Carpacol	Polymer (HPMC)	Curcumin	Propylene glycol	Sodium benzoate	Triethanolamine	Distilled water
Quantity	2gm	2gm	2gm	5ml	0.2ml	q. s	q.s.to make 100 ml

(HPMC = hydroxy propyl methyl cellulose; q.s. =quantity sufficient)

0.2 mg of curcumin (Sigma, St. Lo, USA) was transmitted into an HPMC solution where it was further homogenized. After that, this drug solution was transmitted to a solution of carbopol where it was also homogenized. Then triethanolamine was further added in a quantity sufficient (q.s.) to make neutralization of the ph. The added distilled water made q.s. to 100 ml. finally, the gel was kept at ambient temperature. The prepared curcumin gel was verified and evaluated for microbial growth and activity using procedures including an incubation period of 24 h on an agar plate while associated microbial growth was observed. If no microbial growth and activity were noted, the final curcumin gel preparation was then kept in glass containers for storage in the refrigerator (humidity ranging from 80–85%and 2–10 °C temperature) until the time of use to avoid its subsequent damage [42].

Following SRP procedures, only selected periodontal pockets (pockets diagnosed with stage II grade A, PD of 3–5 mm, and CALof 3-4 mm) [3] within group III were delivered with 2% curcumin gel sub-gingivally using a disposable 2 ml syringe with a blunt needle (bent 130° at the shank). The application of gel was initiated from the base of the pocket to ensure the gel reached the whole pocket depth. This was accomplished by a plastic syringe with a flexible metal needle^1^ with a large gauge (similar to those used for acid etchant application).

This procedure continued until the pocket was filled. Care was taken to apply the gel without traumatizing or damaging the periodontal tissues. After insertion of the curcumin gel, the region was secured with a cotton pack, and the patients were advised not to eat or drink for at least two hours following gel administration. They were also advised not to brush the treated areas or floss /use interproximal cleaning devices for at least twelve hours postoperatively and to avoid eating hard and abrasive foods that could traumatize the gingiva. The treatment was followed-up by weekly recalls after gel application for reinforcement of oral hygiene measurements and subsequent gel reapplication. The curcumin gel was applied weekly for four weeks after completing SRP.

#### Periodontal assessment (primary outcomes)

Periodontal measurements included Primary outcomes; (plaque Index (PI) according to Silness P. Loe H 1964. This index was used to estimate the quantity of plaque relevant to the tooth cervical part as it considers only the difference in the soft deposit thickness with no regard to the plaque coronal extension [43], gingival index** (**GI) according to (Löe and Silness, 1963). This index assesses the gingival condition and records qualitative changes in the gingiva. It usually scores the marginal and interproximal tissues as it measures both the severity and the extension of gingivitis in four tooth sites (papilla of distofacial, papilla of mesiofacial, margins of facial gingiva, and the entire lingual gingival margin of the tooth) [44], probing depth (PD) [45], and clinical attachment level (CAL) [46] recorded at baseline for all groups and again six weeks after periodontal treatment for both group II and group III using a periodontal probe^2^. Measurements were done by a blinded single examiner (SE) who did not know the identity of the groups in which patients were included.

The choice of a six-week recall visit in this study is justified based on several factors, including existing literature and the unique circumstances presented by the COVID-19 pandemic. Firstly, the decision for a shorter follow-up period aligns with findings from a comprehensive review of relevant literature. Numerous studies, as referenced [47–50], have consistently reported satisfactory outcomes with short-term follow-up periods. Secondly, the global constraints imposed by the COVID-19 pandemic necessitated a pragmatic approach to study design. The pandemic reduced accessibility to medical facilities, staffing shortages, and alterations in patient behavior. In light of these challenges, a shorter recall visit interval of six weeks was deemed practical and feasible.

#### Intra-examiner reliability

To achieve intra- examiner calibration, periodontal measurements of 10 patients were performed twice within 2 days before randomized clinical trial (RCT) conduction. Calibration was approved when measurements of PD and CAL in the two instances were within a 1 mm variance in more than 90% of all measurements.

#### Laboratory assessment of PCT level (secondary outcomes)

The salivary samples were collected from all participants at baseline and once more from both group II and group III after six weeks. The samples were kept in sterile Eppendorf tubes and centrifuged at 3000 RPM for approximately 10 min and kept frozen at -20°c until the time of analysis by ELISA test to assess salivary PCT (according to manufacturer instructions)^3^.

**Table 2:** Demographic data and clinical parameters of study groups at baseline

	Group I (n = 18)	Group II (n = 18)	Group III (n = 18)	P
**Sex**				
Male	7 (38.9)	9 (50.0)	4 (22.2)	(χ^2^)p = 0.221
Female	11 (61.1)	9 (50.0)	14 (77.8)	
**Age (years)** (Mean ± SD.)	39.17 ± 5.66	40.83 ± 6.05	40.61 ± 6.51	^F^p= 0.674
**Plaque Index (PI) ** Median (Min. – Max.)	0.50^b^ (0.0–1.0)	3.0^a^ (1.0–3.0)	2.63^a^ (1.0–3.0)	^H^p_1_<0.001* ^H^p_2_<0.001*, ^H^p_3_=0.422,
**Gingival Index (GI) ** Median (Min. – Max.)	0.50^b^ (0.0–1.0)	3.0^a^ (2.0–3.0)	2.63^a^ (2.0–3.0)	^H^p_1_<0.001^*^ ^H^p_2_<0.001^*^, ^H^p_3_=0.308,
**Probing depth (PD)** Mean ± SD.	2.0^b^ ± 0.84	4.65^a^ ± 0.46	4.60^a^ ± 0.22	^F^p_1_<0.001^*^ ^F^p_2_<0.001^*^, ^F^p_3_=0.962,
**Clinical attachment level (CAL) ** Median (Min. – Max.)	0.0^b^ (0.0 ± 0.0)	5.30^a^ (4.20–7.40)	4.75^a^ (4.30–8.0)	^H^p_1_<0.001^*^ ^H^p_2_<0.001^*^, ^H^p_3_=0.742,

**SD: Standard deviation χ**^**2**^: **Chi-square test F**: **F for ANOVA** test **Means** in the same raw with Small common letters are not significant (i.e., Means with Different letters are significant)

**F: F for ANOVA test**, pairwise comparison between every 2 groups were done using **Post Hoc Test (Tukey)**

**H: H for Kruskal Wallis test**, pairwise comparison between every 2 groups were done using **Post Hoc Test (Dunn’s for multiple comparisons test)** p_1_: p-value for comparing **Group I** and **Group II**

p_2_: p-value for comparing **Group I** and **Group III** p_3_: p-value for comparing **Group II** and **Group III**

*: Statistically significant at p ≤ 0.05

### Statistical analysis of the data

The raw data was transferred to the computer and analyzed using IBM SPSS software package version 20.0. (Armonk, NY: IBM Corp). Qualitative data were described using numbers and percentages. The Shapiro-Wilk test was used to verify the normality of distribution and quantitative data was described using range (minimum and maximum), mean, median, and standard deviation. The significance of the obtained results was judged at the 5% level.

The following tests were used: the Chi-square test was used for categorical variables, (to compare between different groups), the Student t-test for parametric quantitative variables, (to compare between two studied groups), the F-test (ANOVA) for parametric quantitative variables, (to compare between more than two groups), the Post Hoc test (Tukey) for pairwise comparisons, the Paired t-test for parametric quantitative variables, (to compare between two periods),

the Mann Whitney test for non-parametric quantitative variables,( to compare between two studied groups), Kruskal Wallis test for non-parametric quantitative variables,( to compare between more than two studied groups), the Post Hoc (Dunn’s multiple comparisons test) for pairwise comparisons, the Wilcoxon signed ranks test for non-parametric quantitative variables, (to compare between two periods), and the Spearman coefficient to correlate between two non-parametric quantitative variables.

### Sample size calculation

Sample size calculation was based on mean superoxide dismutase (SOD) after treatment between the study and control group retrieved from a previous study (Elavarasu et al., 2016) [51]. Using G*power version 3.0.10 to calculate sample size based on the effect size of 0.828, 2-tailed test, α error = 0.05, d power = 90.0% the total calculated sample size was 18 in each group at least.

## Results

The study included fifty-four Patients, twenty males, and thirty-four Females (ranging from thirty to fifty-five years old) with no statistically significant difference between the three studied groups regarding their sex and age distribution (p > 0.05). Table (2).

The results showed a statistically significant difference regarding the clinical indices at baseline of group II and group III as compared to group I (p ≤ 0.05). However, there was no statistically significant difference between group II and group III regarding PI, GI, PD, and CAL at baseline p (> 0.05). Table (2).

**Fig. 2:**
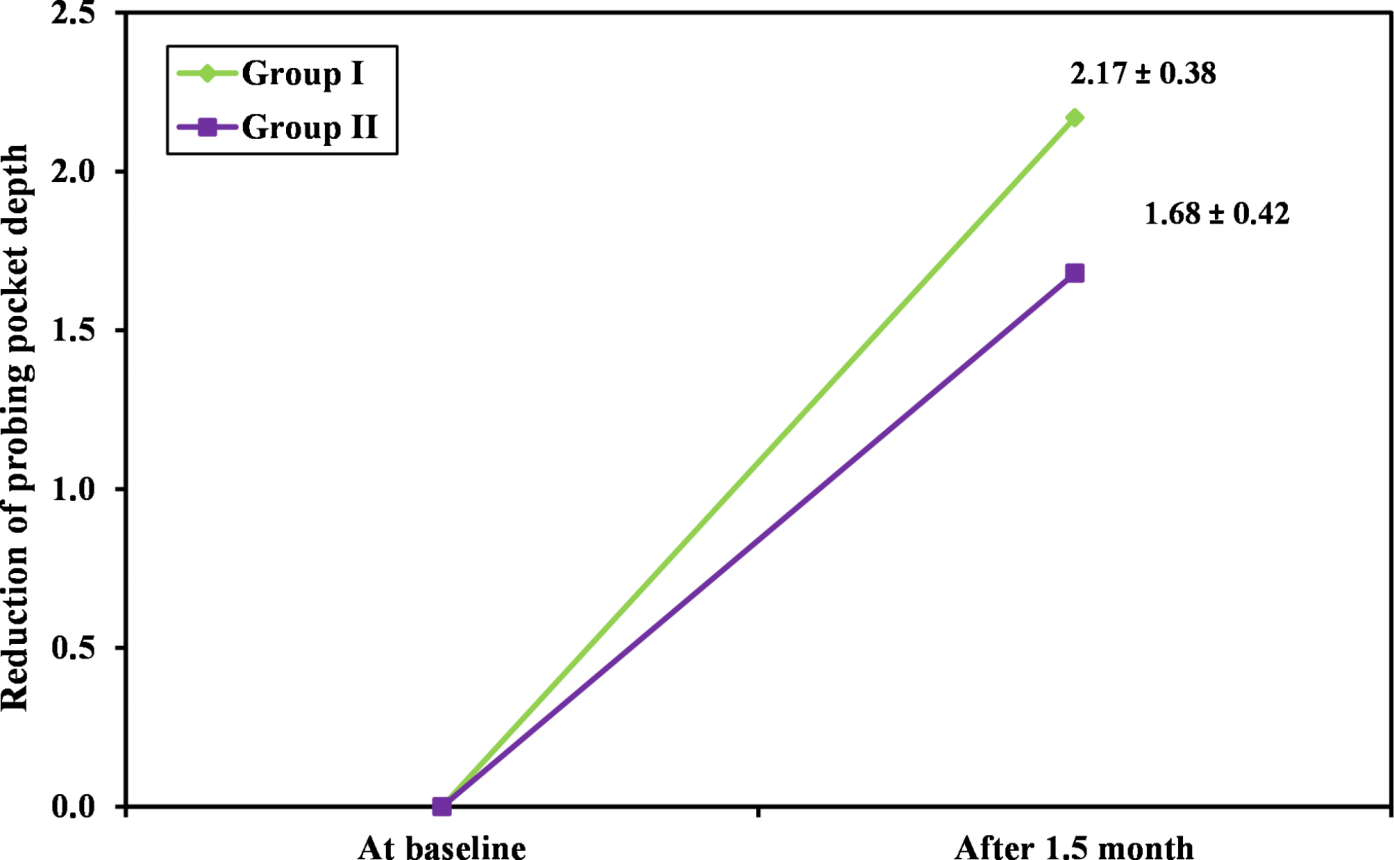
The probing depth reduction in both group II and group III after six weeks

**Fig. 3:**
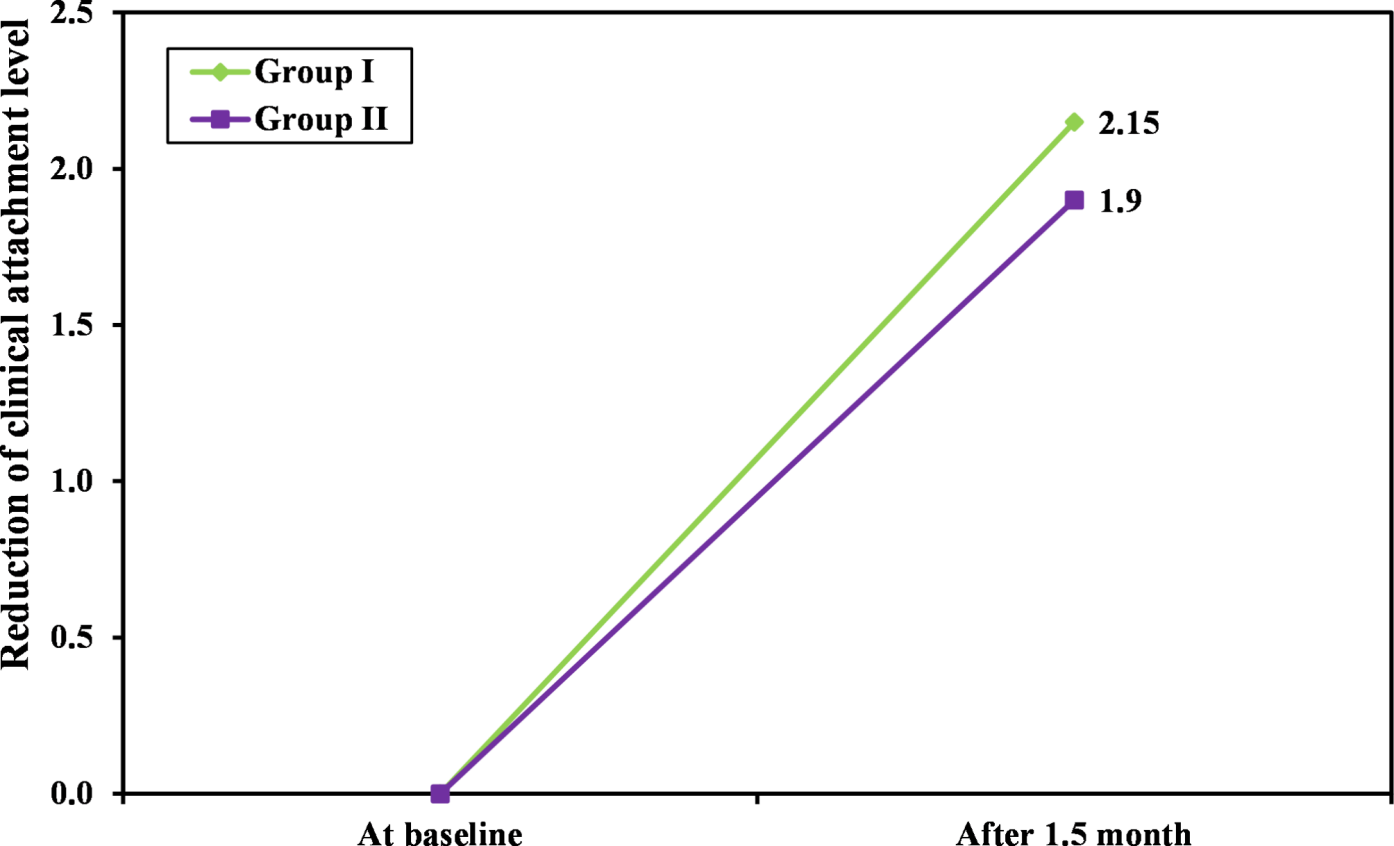
The clinical attachment level gained in both group II and group III after six weeks

Additionally, a statistically significant decrease in all clinical indices of both group II and group III (p ≤ 0.05) was observed six weeks after treatment. Group III showed greater improvement in the PI, PD, and CAL as compared to group II as shown in Table (3) and Figs. (2, 3).

**Table 3:** Clinical parameters of study groups at different time intervals

		Group I (n = 18)	Group II (n = 18)	Group III (n = 18)	Pairwise
**Plaque ** **Index (PI) ** Median (Min. – Max.)	**At baseline**	0.50^b^ (0.0–1.0)	3.0^a^ (1.0–3.0)	2.63^a^ (1.0–3.0)	^H^p_1_<0.001^*^ ^H^p_2_<0.001^*^, ^H^p_3_=0.422,
	**After 1.5 month**	0.50^b^ (0.0–1.0)	1.0^a^ (0.0–1.0)	0.75^b^ (0.0–1.0)	^H^p_1_<0.001^*^ ^H^p_2_=0.580, ^H^p_3_=0.003^*^,
	**Test of sig. (p**_**0**_ **)**		Z = 3.831^*^ (< 0.001^*^)	Z = 3.834^*^ (< 0.001^*^)	
**Gingival Index (GI)** Median (Min. – Max.)	**At baseline**	0.50^b^ (0.0–1.0)	3.0^a^ (2.0–3.0)	2.63^a^ (2.0–3.0)	^H^p_1_<0.001^*^ ^H^p_2_<0.001^*^, ^H^p_3_=0.308,
	**After 1.5 month**	0.50^b^ (0.0–1.0)	1.0^a^ (–)	1.0^a^ (0.75– 1.0)	^H^p_1_<0.001^*^ ^H^p_2_=0.001^*^, ^H^p_3_=0.247,
	**Test of sig. (p**_**0**_ **)**		Z = 3.898^*^ (< 0.001^*^)	Z = 3.775^*^ (< 0.001^*^)	
**Probing depth (PD)** Mean ± SD.	**At baseline**	2.0^b^ ± 0.84	4.65^a^ ± 0.46	4.60^a^ ± 0.22	^F^p_1_<0.001^*^ ^F^p_2_<0.001^*^, ^F^p_3_=0.962,
	**After 1.5 month**	2.0^b^ ± 0.84	2.97^a^ ± 0.63	2.43^b^ ± 0.36	^F^p_3_<0.001^*^ ^F^p_2_=0.122, ^F^p_1_=0.037^*^,
	**Test of sig. (p**_**0**_ **)**		t = 16.899^*^ (< 0.001^*^)	t = 24.522^*^ (< 0.001^*^)	
**Clinical attachment level (CAL) ** Median (Min. – Max.)	**At baseline**	0.0^b^ (0.0 ± 0.0)	5.30^a^ (4.2–7.40)	4.75^a^ (4.30–8.0)	^H^p_1_<0.001^*^ ^H^p_2_<0.001^*^, ^H^p_3_=0.742,
	**After 1.5 month**	0.0^b^ (0.0±. 0.0)	3.40^a^ (2.0–6.10)	2.60^b^ (1.80–5.6)	^H^p_1_<0.001^*^ ^H^p_2_=0.059, ^H^p_3_=0.039^*^,
	**Test of sig. (p**_**0**_ **)**		Z = 3.733^*^ (< 0.001^*^)	Z = 3.729^*^ (< 0.001^*^)	

**SD: standard deviation Z: Wilcoxon signed ranks test t: paired t-test**

**Means** in the same raw with common letters are not significant (i.e., Means with Different letters are significant)

**F**: **F for ANOVA test**, pairwise comparison between every 2 groups were done using **Post Hoc Test (Tukey)**

**H: H for Kruskal Wallis test**, pairwise comparison between every 2 groups were done using **Post Hoc Test (Dunn’s for multiple comparisons test)**

p_0_: p-value for comparing between baseline and after 1.5 month p_1_: p-value for comparing **Group I** and **Group II**

p_2_: p-value for comparing **Group I** and **Group III** p_3_: p-value for comparing **Group II** and **Group III** *: Statistically significant at p ≤ 0.05

**Fig. 4:**
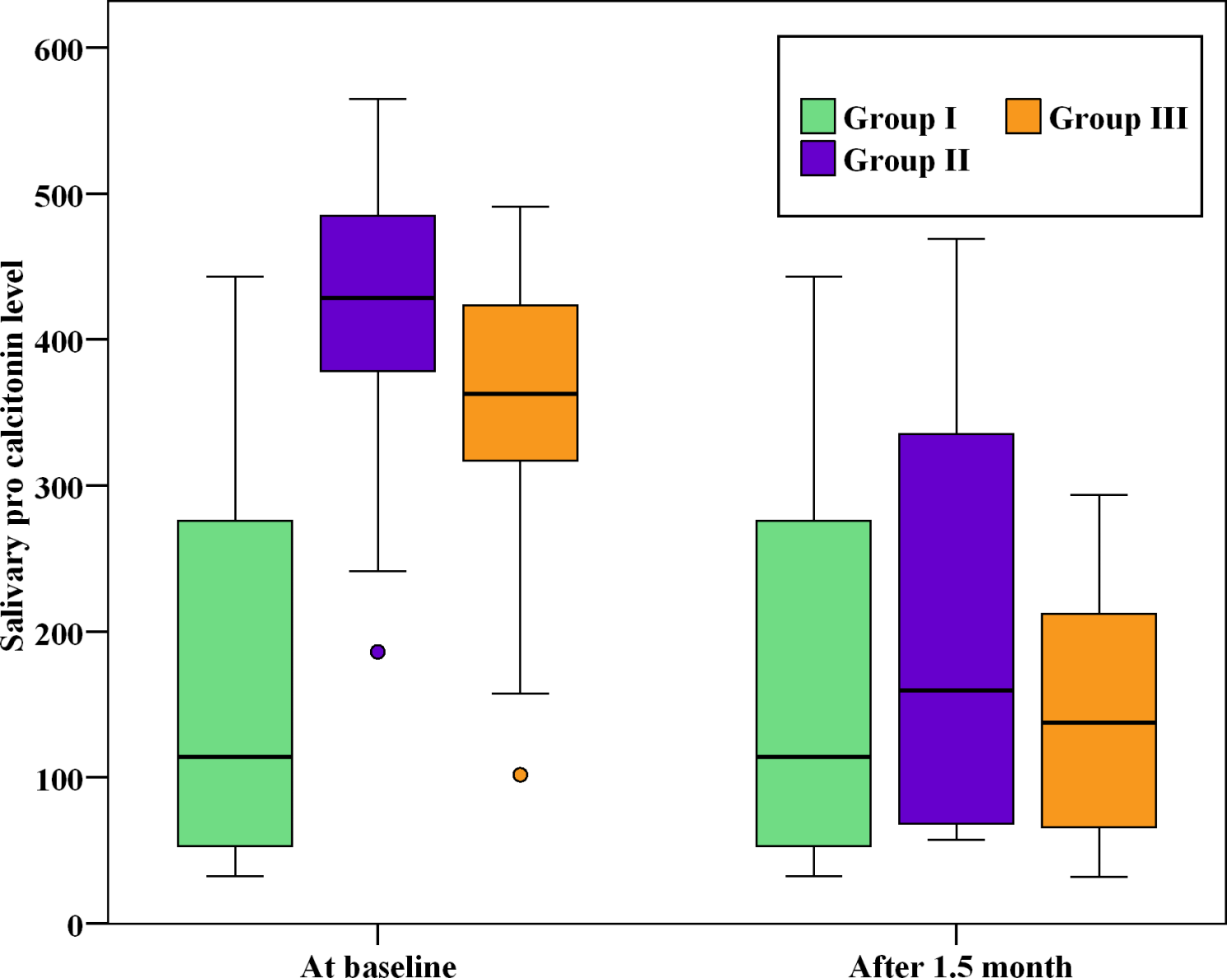
Box plot showing the comparison between the three studied groups according to salivary PCT level at different time intervals

While there wasn’t a statistically significant difference between group II and group III regarding salivary PCT level (p > 0.05) at baseline, there was a statistically significant difference between group II and group III compared to group I (p ≤ 0.05). Six weeks after treatment, there was a statistically significant decrease in PCT levels of both groups II and III (p ≤ 0.05). However, regarding salivary PCT levels, there wasn’t a statistically significant difference between group II and group III after treatment (p > 0.05) as shown in Fig. (4).

Table (4) shows the salivary PCT level of study groups at different time intervals. At baseline, there wasn’t a statistically significant difference between group I and group II (p > 0.05). However, there was a significant statistical difference between groups I and group II, and group III (p ≤ 0.05). At six weeks after treatment, there was a significant statistical decrease in both groups I and group II PCT levels (p ≤ 0.05). However, there was an insignificant statistical difference in PCT levels among the study groups after treatment (p > 0.05).

**Table 4:** Salivary PCT level of study groups at different time intervals

		Group III (n = 18)	Group II (n = 18)	Group I (n = 18)	H (p)
Salivary pro calcitonin level (pg./ml ) Median (Min. – Max.)	**At baseline**	428.5a (186.1–564.8)	362.6a (101.9–490.7)	114.1b (32.30–443.0)	21.406* (< 0.001*)
	**After 1.5 month**	159.8a (57.59–468.7)	137.9a (32.10–32.1)	114.1a (32.30–443.0)	1.507 (0.471)
	**Z (p0)**	3.724* (< 0.001*)	3.724* (< 0.001*)		

Medians with common letters are insignificant in the same raw (i.e. Medians with dissimilar letters are significant)

Z: Wilcoxon signed ranks test

H: H for Kruskal Wallis test

P: p-value for comparing the three studied groups

p0: p-value for comparing between baseline and after 1.5 month

*: Statistically significant at p ≤ 0.05

## Discussion

The goal of periodontal treatment is to eliminate the bacterial plaque and the associated factors enhancing its accumulation. Despite SRP being the routine therapeutic modality [52], The concomitant administration of local drug delivery is associated with improved the periodontal tissue condition with less drug resistance, less systemic side effects, and enhanced penetration of the drug in the diseased sites resulting in the elimination of harmful pathogenic bacteria.

The use of natural herbal products in dentistry has become ever-increasing. This can be attributed to their low cost, limited side effects, and inherent availability [53]. Among these herbal products is curcumin which has been proven to exhibit antiseptic, anti-inflammatory, antimicrobial, and antioxidant properties with no any adverse effects have been reported in this study during the whole study period. This study aims to assess the effect of local drug delivery via curcumin gel as a natural herbal product in the treatment of stage II grade A periodontitis [54].

Furthermore, the assessment of salivary biomarkers such as PCT has become an important part of laboratory diagnosis for the prediction of periodontitis [55]. Most cases of microbial infections involving periodontitis are associated with elevated PCT levels [55]. Moreover, serum PCT significantly correlates with salivary levels at baseline as well as after periodontal therapy as described by RENJITH, et al., (2021). Hence, salivary PCT offers many advantages like ease of collection and handling, making it an excellent alternative biomarker to its serum counterpart [56].

Regarding the results of this study, all baseline recorded clinical parameters; GI, PI, CAL, and PD showed insignificant variance among both the study group (group III) and positive control group (group II), whereas there was a statistically significant variance among the two groups of periodontitis (group II and III) as opposed to the clinically healthy control group. Furthermore, there was a significant improvement in all clinical indices in both group II and group III at six weeks after treatment. This can be explained due to SRP being considered the gold standard for mechanical therapy in the treatment of periodontitis [57]. However, group III showed a statistically significant better reduction in the clinical indices as compared to group II after treatment.

On the other hand, regarding the salivary PCT baseline levels in the study, there wasn’t a statistically significant variance between group II and group III. However, the PCT level in both groups was significantly elevated as opposed to group I. After six weeks of recall appointments and follow-ups, the levels of salivary PCT reduced significantly in both groups II and group III and dropped to that of group I as there wasn’t a statistically significant difference between the three groups after treatment.

In this study, there was a significant improvement in all clinical indices in both group II and group III after treatment. This can be explained due to SRP being considered the gold standard [58]for mechanical therapy in the treatment of periodontitis. However, group III showed a statistically significantly better reduction in the clinical indices as compared to group II after treatment. These results can be attributed to the effect of the curcumin gel in restoring normal gingival anatomy and tone due to its known properties as an antioxidant, anti-inflammatory, antimicrobial, immunostimulant, and antiseptic. Moreover, it has a potential effect on accelerating wound healing. Therefore, curcumin may be an optimistic local drug delivery agent that can be used in conjunction with SRP as a treatment strategy to overcome the drawbacks of other chemicals and compounds.

The results of the study are consistent with those of Behal, et al., (2011) and Bhatia, et al.,(2014) who evaluated a local drug delivery containing curcumin gel with SRP compared to SRP alone and found a significant reduction in all clinical indices with greater improvement was seen in all indices in the curcumin group as opposed to the SRP group with better restoring of gingival health [16, 59].

Likewise, Anitha, et al., (2015), Hugar, et al., (2016), and Siddharth, et al., (2020) in their studies comparing curcumin as local drug delivery and chlorohexidine ( which is a gold standard), found better improvement in both groups with a greater reduction in all measured parameters in curcumin group [14, 60, 61]. P. L. Ravishankar, et al., (2017), evaluated the effect of curcumin and ornidazole gel in treating chronic periodontitis, they found that the curcumin group showed a significant decrease in all measured clinical parameters when compared to the ornidazole group [62].

Regarding the baseline salivary PCT levels in this study, there wasn’t a statistically significant difference between group II and group III and the level in both groups was significantly higher as opposed to group I. This could be attributed to the production of PCT in response to inflammation with the simultaneous release of endotoxin from nearly all body tissues. As per the literature, Periodontitis is an infectious disease caused by Gram-negative pathogenic bacteria generating endotoxins with subsequent enhancement of PCT secretion in saliva [63, 64].

However, the results of one study indicated that patients with periodontitis and with periodontitis associated with coronary heart disease had higher serum and salivary Galectin-3 levels compared with CHD patients and healthy controls [23]. The results of another study indicated that periodontal treatment performed with full mouth scaling and root planning (FMSRP) was more efficacious in reducing clinical parameters and serum NT-proBNP and related early CVD risk biomarkers in patients with periodontitis at 6-month follow-up performed with FM-SRP [65].

After six weeks of recall appointments and follow-ups, the levels of salivary PCT reduced significantly in both groups II and group III and dropped to that of group I as there wasn’t a statistically significant difference between the three groups after treatment. The reduction of salivary PCT levels after treatment can be attributed in -part to the effective SRP as it is considered the gold standard in the treatment of periodontitis [58]. In addition to SRP, the oral hygiene protocol was followed by patients in both groups II and group III which resulted in the reduction of the sub gingival bacterial load and subsequent reduction of PCT levels. However, there was no significant difference in PCT level between group II and group III after treatment.

These results are in line with Hendek, et al., (2015), which found an increase in salivary PCT level in periodontitis and positively correlated the level to clinical indices such as PD, CAL, and GI. RENJITH, et al., (2021), assessed salivary and serum PCT in systemically healthy periodontitis patients before and after SRP. They found a significant reduction in saliva and serum PCT levels one month after SRP indicating the impact of periodontal therapy on systemic inflammation. They also concluded that salivary PCT is positively correlated with serum PCT, so it can be considered as an alternative biomarker to its serum counterpart [56]. Moreover, Cynthia, et al.,(2022), concluded that the expression of procalcitonin in serum was increased to eightfold in the periodontitis group with diabetes in comparison to the healthy group, which showed that periodontal disease can cause the release of procalcitonin [66].

Finally, this study demonstrated a statistically significant positive correlation between salivary PCT levels and clinical indices in the periodontitis patients (group II and group III). This correlation was found in the total patients in both group II and group III. And this statistically positive significant correlation was only in the total patient population and not in the individual groups as the sample size was too small in each group.

These results could be explained by the common contributing factors that caused elevation in both the clinical indices and salivary PCT levels before treatment such as poor oral hygiene, accumulation of plaque and calculus with subsequent elevation of oral microbial flora, associated periodontal destruction with the secretion of inflammatory biomarkers (including salivary PCT) [67, 68] and the release of cytokines at tissue-injury sites [69].

Although there was a statistically significant decrease in clinical parameters and salivary PCT levels after treatment, there wasn’t a statistically significant correlation between clinical indices and salivary PCT levels. This is in agreement with Bassim et al.(2008) who observed a reduction in salivary PCT levels 3 months after periodontal therapy, but it was not statistically significant [67].

So, Repeated clinical applications of curcumin gel as an adjunct to standard SRP have demonstrated a substantial outcome on all assessed clinical indices between the selected pockets with greater improvement as compared to SRP only as well as excellent restitution of attachment loss, and repair of periodontal tissue even with further deep periodontal pockets PCT levels in saliva in periodontal health and disease were estimated and results suggested that its levels increased progressively in saliva with the severity of the periodontal disease.PCT concentration in saliva was found to be highest in both periodontitis groups and lowest in the periodontally healthy group. After the periodontal treatment, the level of PCT in saliva was significantly decreased in periodontitis patients.

### Conclusion and limitations

#### Clinical significance

This research work was carried out to test curcumin gel which is a natural herbal product as a promising local drug delivery agent with the advantages of inherent availability, low cost, and fewer side effects. Additionally, this research evaluated salivary PCT as an inflammatory biomarker and the efficacy of applied curcumin gel on its levels during the treatment of periodontitis patients.

As demonstrated in the study, curcumin gel has been proven to be an encouraging local drug delivery with a positive influence in all measured clinical indices with no reported side effects. Additionally, there was a positive correlation between PCT and clinical indices before treatment due to common contributing factors including the presence of the subgingival bacteria and subsequent inflammatory response. Therefore, it can be concluded that PCT can be used as an inflammatory biomarker in the assessment and diagnosis of periodontal disease.

#### Limitations of this study

However, there were some limitations of this study due to short follow-up period and small size so further studies with long duration and larger sample size are required .Also other biomarkers which are more relevant to the systemic inflammatory diseases should be studied such as Galectin-3, and NT-proBNP.

## Data Availability

The datasets used during the current study are available from the corresponding author upon request.

## Footnotes

1 Acid etchant pre-bent needle tip with 25 G and 0.5 mm.

2 A graduated 1 mm UNC 15 probe.

3 Human Procalcitonin ELISA Kit (*Cat*. No E0977Hu) was used for the assessment of PCT levels in the collected samples according to manufacturer instructions. Bioassay Technology laboratory, China.
